# Supplementary material for: Splice-Junction-Based Mapping of Alternative Isoforms in the Human Proteome
Source: Cell Rep. Author manuscript; Available in PMC 2020 Jan 15. (PMC6961840; doi:10.1016/j.celrep.2019.11.026)
Supplement: 3 [file NIHMS1546469-supplement-3.zip › DF2/PXD0010154/Thyroid-46-Q99996-QLAQNGNENQGEVEEQTFK.pdf]

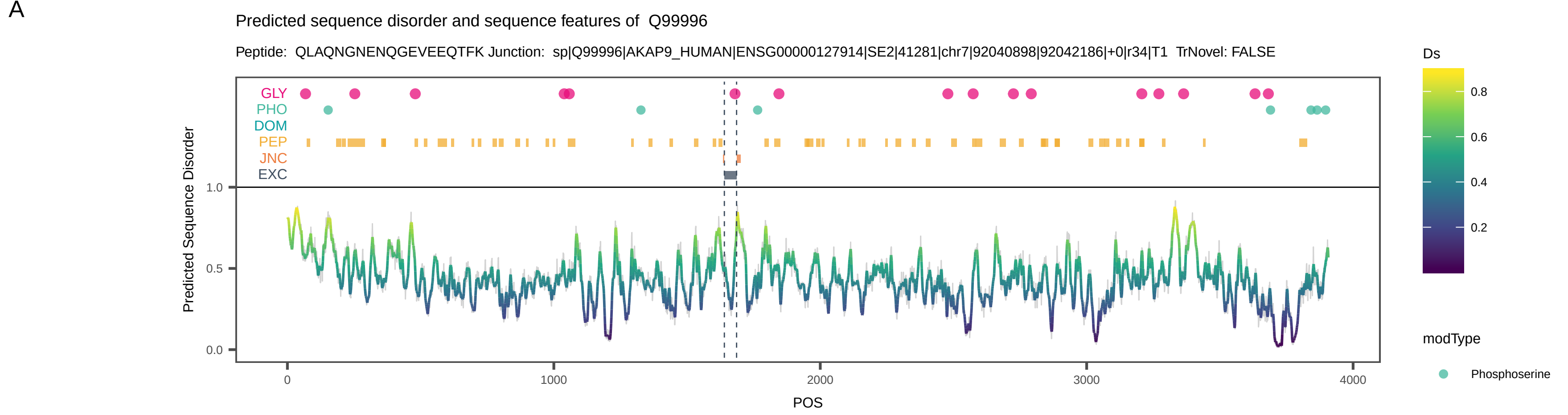
A
Predicted sequence disorder and sequence features of Q99996
Peptide: QLAQNGNENQGEVEEQTFK Junction: sp|Q99996|AKAP9_HUMAN|ENSG00000127914|SE2|41281|chr7|92040898|92042186|+0|r34|T1 TrNovel: FALSE
GLY
PHO
DOM
PEP
JNC
EXC
Predicted Sequence Disorder
1.0
0.5
0.0
0
1000
2000
3000
4000
POS
Ds
0.8
0.6
0.4
0.2
modType
Phosphoserine

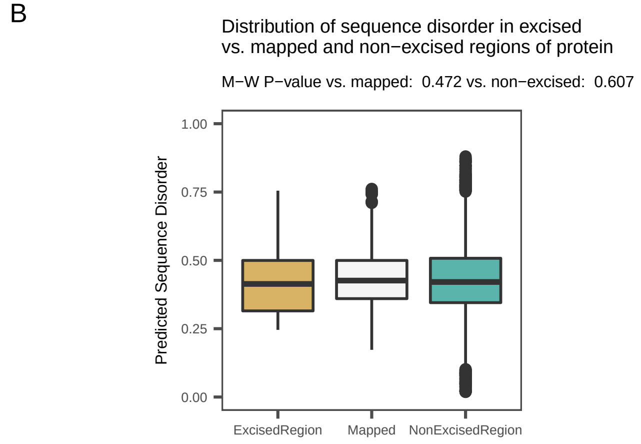
B
Distribution of sequence disorder in excised
vs. mapped and non−excised regions of protein
M−W P−value vs. mapped: 0.472 vs. non−excised: 0.607
Predicted Sequence Disorder
1.00
0.75
0.50
0.25
0.00
ExcisedRegion
Mapped
NonExcisedRegion

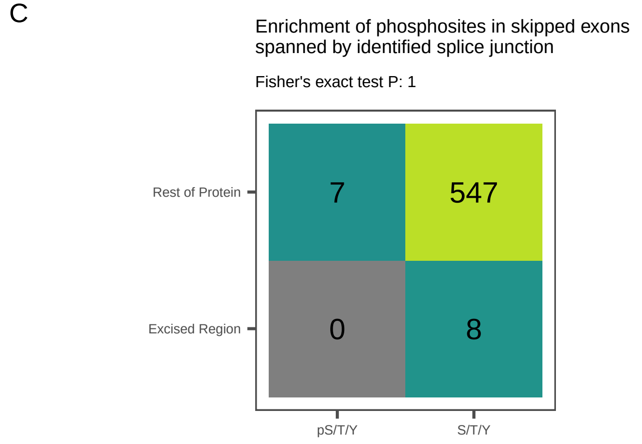
C
Enrichment of phosphosites in skipped exons
spanned by identified splice junction
Fisher's exact test P: 1
Rest of Protein
Excised Region
7
547
0
8
pS/T/Y
S/T/Y
